# CB1 receptor and psychosis: friend, foe, or both

**DOI:** 10.3389/fpsyt.2025.1630080

**Published:** 2025-07-22

**Authors:** Jagadeesh S. Rao, Maria Alejandra Tangarife, Diego A. Rodríguez-Soacha, Ram Mukunda

**Affiliations:** IGC Pharma Inc, Potomac, MD, United States

**Keywords:** Alzheimer’s disease, anandamide, CB1R, psychosis, CB2 receptor, cannabis, schizophrenia

## 1 Introduction

Psychosis is a core issue in Schizophrenia (SZ), whereas in Alzheimer’s disease (AD), the prevalence of psychosis, particularly hallucinations and delusions, is approximately 40 to 60% (1). The onset of psychosis varies in SZ and AD but shares similar structural, neurochemical changes, and genetic associations (2). Radiological and pathological studies reveal a common pattern of limbic system involvement in both SZ and AD, with predominant atrophy in the medial temporal regions. Both conditions also share core neurochemical disturbances, particularly affecting the dopaminergic and cholinergic systems (3). Both SZ and AD are characterized by progressive cognitive decline and structural brain changes, including white matter abnormalities, which are key features of both disorders (2). Early cannabis use, particularly before ages 15–18, has been consistently linked to an increased risk of developing psychotic symptoms, with the risk rising in a dose-dependent manner based on frequency and THC potency (4). A landmark UK study involving 780 adults (410 with a first episode of psychosis and 370 matched controls) found that daily use of high-potency cannabis (>15% THC) was associated with a fivefold increased risk of psychosis. In contrast, individuals who used low-potency hash (<5% THC) did not exhibit an increased risk of psychotic symptoms (5). In a longitudinal cohort of 1,037 individuals followed from childhood to midlife, persistent cannabis use was linked to a measurable decline in IQ across development (6). Additionally, individuals homozygous for the Val allele of the catechol-O-methyltransferase (COMT) gene (Val/Val genotype) demonstrated an increased vulnerability to cannabis-induced psychosis, highlighting a gene-environment interaction that may modulate the neuropsychiatric consequences of cannabis exposure (7). Current preclinical and clinical evidence indicates that cannabis exposure during the vulnerable period of adolescence is associated with persistent behavioral and neurocognitive impairments later in life, resembling features of schizophrenia-related disorders. These adverse effects are influenced by user-specific factors, such as sex, genetic predisposition, duration, and intensity of exposure, as well as drug-specific variables, including THC: CBD ratios and THC potency. Similarly, in rodent models where exposure was limited to adolescence and outcomes were assessed in adulthood, the observed effects varied depending on dose, timing, and strain, though most studies report detrimental consequences of adolescent THC exposure (8).

## 2 Role of the endocannabinoid system in psychosis

The endocannabinoid system (ECS) comprises two main cannabinoid receptors (CBRs): CB1Rs, predominantly located in the central nervous system (CNS), and CB2Rs, primarily found in peripheral tissues and the CNS. It also includes two principal endogenous ligands, anandamide (AEA) and 2-arachidonoylglycerol (2-AG), as well as their key synthesizing enzymes, N-acyl phosphatidylcholine phospholipase D and diacylglycerol lipase. Degradation of these ligands is mediated by fatty acid amide hydrolase (FAAH) and monoacylglycerol lipase (MAGL) (9). Elevated cerebrospinal fluid (CSF) anandamide levels have been observed in individuals exhibiting early signs of schizophrenic symptoms compared to healthy controls. Notably, within this high-risk population, lower anandamide concentrations are associated with an increased risk of transitioning to psychosis. These findings suggest that heightened anandamide signaling may represent an endogenous compensatory mechanism aimed at mitigating psychotic symptom progression (9, 10). Among those with psychosis, lower severity of symptoms is linked to higher levels of anandamide in the cerebrospinal fluid (10). In individuals with SZ, chronic cannabis use is not associated with elevated CSF AEA levels, implying that cannabis may disrupt the brain’s innate protective mechanisms against psychosis and contribute to its pathogenesis in susceptible individuals (11). Exploratory findings from PET imaging studies suggest that lower brain CB1 receptor availability is linked to more severe symptoms and greater cognitive impairment in male patients compared to controls (12). Another PET cohort study has revealed a significant reduction in the CB1R levels in the amygdala, caudate, posterior cingulate cortex, hippocampus, hypothalamus, and insula of male SZ patients compared to controls. Further, medicated SZ patients with haloperidol and risperidone have increased levels of CB1R binding compared to the unmedicated SZ patients (13). While the exact mechanism behind reduced CB1 receptor levels in psychosis is not fully understood, research indicates that synthetic CB1R agonists or AEA analogues can trigger CB1R internalization through endocytosis, leading to decreased receptor expression on the cell surface. In patients with first-episode psychosis (FEP) who do not use cannabis, elevated endogenous AEA levels may similarly promote CB1R internalization, potentially explaining the observed reduction in receptor availability (11). Supporting this possibility, studies in mice lacking FAAH, an enzyme responsible for anandamide degradation, have shown that AEA administration leads to region-specific reductions in CB1 receptor levels (14). However, a similar mechanism occurring in humans is not clear. Like SZ pathology, a network meta-analysis suggests that individuals with AD may exhibit reduced CB1R expression, accompanied by elevated levels of 2-AG and increased expression of its degrading enzyme MAGL (15). However, this imbalance was not compared against psychosis in AD. Selective dysregulation of the peripheral ECS was observed in SZ, marked by reduced DNA methylation at the CNR1 gene promoter that encodes CB1R in peripheral blood mononuclear cells (PBMCs), a change not seen in bipolar or major depressive disorder. This finding was validated in a prenatal MAM-exposed schizophrenia animal model, showing increased CBR1 expression and reduced promoter methylation in the prefrontal cortex (16). Further preclinical and clinical studies indicate that perinatal exposure to THC disrupts the regulation of CB1 and dopamine (Drd2) receptor genes in the prefrontal cortex of rats, causing increased gene expression and reduced DNA methylation. These molecular changes are linked to behavioral abnormalities, such as social withdrawal and cognitive deficits in adulthood, which were prevented by treatment with cannabidiol during puberty. Similar epigenetic alterations in DRD2 were also found in SZ patients, suggesting that early-life THC exposure may contribute to SZ risk through lasting changes in the dopamine and endocannabinoid systems, and that cannabidiol may help reverse these effects (17). In support of these findings, a recent study examined DNA methylation of the CBR1 and DRD2 genes in saliva samples from psychotic patients. Results showed higher methylation levels at these genes in psychotic subjects compared to healthy controls, with levels being lower in THC-using patients, particularly DRD2, resembling those of non-psychotic controls. These findings highlight CNR1 and DRD2 methylation as potential epigenetic biomarkers of psychosis, though this may not apply to active THC users since THC can modify the DNA methylation (18).

**Figure 1:**
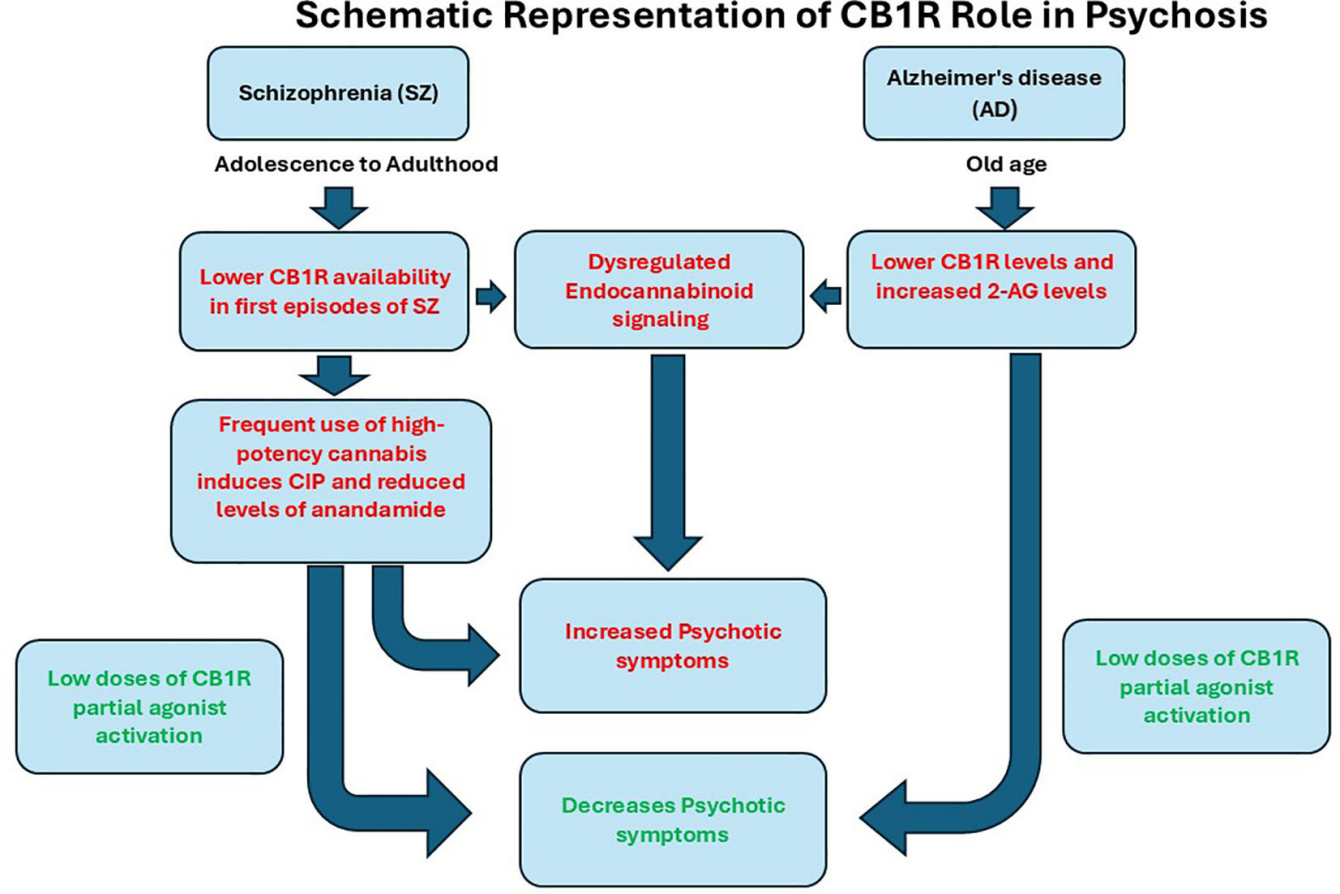
Schematic representation of CB1R role in Psychosis in Schizophrenia and Alzheimer’s disease. Altered endocannabinoid systems have been observed in both SZ and AD, with loss of CB1R and altered endocannabinoid levels in SZ and AD. Decreased expression of CB1R and reduced levels of anandamide were associated with an increase in psychotic symptoms in SZ. Similar CB1R changes were observed in AD with increased endocannabinoid levels of AEA. The stimulation of CB1R by its partial agonist has beneficial effects, alleviating psychotic symptoms.

In an open-label study involving a small cohort of SZ patients, low-dose treatment with the CB1 partial agonist dronabinol over eight weeks was associated with a reduction in core psychotic symptoms (19). Similar effects have been observed in AD, where open-label studies show that two different studies revealed a decrease in hallucinations and delusion symptoms, and other beneficial effects with low doses of cannabis (20, 21). CB1R acts as a friend/valuable for the normal maintenance of synaptic plasticity (**Figure 1**). It appears that loss of CB1R may be associated with psychiatric symptoms, and partial activation of CB1R with low doses of THC may be beneficial in both chronic conditions. Overstimulation of CB1R by high potency heavy use may lead to downregulation of CB1R and may result in CIP. CB1 receptors play a key role in maintaining the balance between excitatory and inhibitory neurotransmission via endogenous cannabinoids such as AEA. During adolescence, cannabis exposure may disrupt glutamatergic signaling, a process essential for synaptic pruning in the prefrontal cortex, potentially impairing normal brain development (22). Evidence of declining CB1R during AD may contribute to hallucinations and delusions (23). Fine-tuning of the CB1R receptor may correct aberrant signaling of CB1R. CB1R has a dual role in modulating the response to the internal endocannabinoid as well as to external cannabis use. Imbalance in CB1R function is associated with behavioral changes seen in both conditions. Over the past two decades, growing research has linked frequent cannabis use during adolescence to mild cognitive impairments. However, it remains unclear whether these effects persist after prolonged abstinence. Inconsistencies in neuroimaging findings further complicate conclusions, underscoring the need for larger, better-controlled studies to clarify long-term brain and behavioral outcomes (24). More validated randomized placebo studies of CB1R partial agonists are warranted to establish efficacy in both SZ and AD.

## 3 Conclusions

Imbalance in CB1R and its endogenous agonist may play a role in developing psychosis symptoms in SZ and AD. High-potency cannabis use during adolescence worsens this disruption, increasing psychosis risk and emphasizing the need for targeted therapies and further clinical studies. In AD and SZ, low-dose CB1R activation via a CB1R partial agonist may alleviate psychotic symptoms. Thus, CB1R can act as both a risk factor and a therapeutic target, depending on context, timing, and dosage.

## References

[1] DeMichele-Sweet MAA Weamer EA Klei L Vrana DT Hollingshead DJ Seltman HJ. Genetic risk for schizophrenia and psychosis in Alzheimer disease. Mol Psychiatry. (2018) 23:963–72. doi: 10.1038/mp.2017.81, PMID: , PMID: 28461698 PMC5668212

[2] Ohi K Fujikane D Shioiri T. Genetic overlap between schizophrenia spectrum disorders and Alzheimer's disease: Current evidence and future directions - An integrative review. Neurosci Biobehav Rev. (2024) 167:105900. doi: 10.1016/j.neubiorev.2024.105900, PMID: , PMID: 39298993

[3] White KE Cummings JL. Schizophrenia and Alzheimer's disease: clinical and pathophysiologic analogies. Compr Psychiatry. (1996) 37:188–95. doi: 10.1016/S0010-440X(96)90035-8, PMID: , PMID: 8732586

[4] Stuyt E. The problem with the current high potency THC marijuana from the perspective of an addiction psychiatrist. Mo Med. (2018) 115:482–6., PMID: , PMID: 30643324 PMC6312155

[5] Di Forti M Marconi A Carra E Fraietta S Trotta A Bonomo M. Proportion of patients in south London with first-episode psychosis attributable to use of high potency cannabis: a case-control study. Lancet Psychiatry. (2015) 2:233–8. doi: 10.1016/S2215-0366(14)00117-5, PMID: , PMID: 26359901

[6] Meier MH Caspi A Ambler A Harrington H Houts R Keefe RS. Persistent cannabis users show neuropsychological decline from childhood to midlife. Proc Natl Acad Sci U.S.A. (2012) 109:E2657–2664. doi: 10.1073/pnas.1206820109, PMID: , PMID: 22927402 PMC3479587

[7] Caspi A Moffitt TE Cannon M McClay J Murray R Harrington H. Moderation of the effect of adolescent-onset cannabis use on adult psychosis by a functional polymorphism in the catechol-O-methyltransferase gene: longitudinal evidence of a gene X environment interaction. Biol Psychiatry. (2005) 57:1117–27. doi: 10.1016/j.biopsych.2005.01.026, PMID: , PMID: 15866551

[8] Stark T Di Martino S Drago F Wotjak CT Micale V. Phytocannabinoids and schizophrenia: Focus on adolescence as a critical window of enhanced vulnerability and opportunity for treatment. Pharmacol Res. (2021) 174:105938. doi: 10.1016/j.phrs.2021.105938, PMID: , PMID: 34655773

[9] Manseau MW Goff DC. Cannabinoids and schizophrenia: risks and therapeutic potential. Neurotherapeutics. (2015) 12:816–24. doi: 10.1007/s13311-015-0382-6, PMID: , PMID: 26311150 PMC4604190

[10] Giuffrida A Leweke FM Gerth CW Schreiber D Koethe D Faulhaber J. Cerebrospinal anandamide levels are elevated in acute schizophrenia and are inversely correlated with psychotic symptoms. Neuropsychopharmacology. (2004) 29:2108–14. doi: 10.1038/sj.npp.1300558, PMID: , PMID: 15354183

[11] Leweke FM Giuffrida A Koethe D Schreiber D Nolden BM Kranaster L. Anandamide levels in cerebrospinal fluid of first-episode schizophrenic patients: impact of cannabis use. Schizophr Res. (2007) 94:29–36. doi: 10.1016/j.schres.2007.04.025, PMID: , PMID: 17566707

[12] Borgan F Laurikainen H Veronese M Marques TR Haaparanta-Solin M Solin O. *In vivo* availability of cannabinoid 1 receptor levels in patients with first-episode psychosis. JAMA Psychiatry. (2019) 76:1074–84. doi: 10.1001/jamapsychiatry.2019.1427, PMID: , PMID: 31268519 PMC6613300

[13] Ranganathan M Cortes-Briones J Radhakrishnan R Thurnauer H Planeta B Skosnik P. Reduced brain cannabinoid receptor availability in schizophrenia. Biol Psychiatry. (2016) 79:997–1005. doi: 10.1016/j.biopsych.2015.08.021, PMID: , PMID: 26432420 PMC4884543

[14] Falenski KW Thorpe AJ Schlosburg JE Cravatt BF Abdullah RA Smith TH. FAAH-/- mice display differential tolerance, dependence, and cannabinoid receptor adaptation after delta 9-tetrahydrocannabinol and anandamide administration. Neuropsychopharmacology. (2010) 35:1775–87. doi: 10.1038/npp.2010.44, PMID: , PMID: 20357755 PMC2895947

[15] Liu Y Xing H Zhang Y Song Y. The endocannabinoid system in alzheimer's disease: A network meta-analysis. J Neurosci Res. (2024) 102:e25380. doi: 10.1002/jnr.v102.9, PMID: , PMID: 39245959

[16] D'Addario C Micale V Di Bartolomeo M Stark T Pucci M Sulcova A. A preliminary study of endocannabinoid system regulation in psychosis: Distinct alterations of CNR1 promoter DNA methylation in patients with schizophrenia. Schizophr Res. (2017) 188:132–40. doi: 10.1016/j.schres.2017.01.022, PMID: , PMID: 28108228

[17] Di Bartolomeo M Stark T Maurel OM Iannotti FA Kuchar M Ruda-Kucerova J. Crosstalk between the transcriptional regulation of dopamine D2 and cannabinoid CB1 receptors in schizophrenia: Analyses in patients and in perinatal Delta9-tetrahydrocannabinol-exposed rats. Pharmacol Res. (2021) 164:105357. doi: 10.1016/j.phrs.2020.105357, PMID: , PMID: 33285233

[18] Di Bartolomeo M Cernanova A Petrusova V Di Martino S Hodosy J Drago F. DNA methylation at cannabinoid type 1 and dopamine D2 receptor genes in saliva samples of psychotic subjects: Is there an effect of Cannabis use? Pharmacol Res. (2024) 208:107343. doi: 10.1016/j.phrs.2024.107343, PMID: , PMID: 39127265

[19] Schwarcz G Karajgi B McCarthy R. Synthetic delta-9-tetrahydrocannabinol (dronabinol) can improve the symptoms of schizophrenia. J Clin Psychopharmacol. (2009) 29:255–8. doi: 10.1097/JCP.0b013e3181a6bc3b, PMID: , PMID: 19440079

[20] Shelef A Barak Y Berger U Paleacu D Tadger S Plopsky I. Safety and efficacy of medical cannabis oil for behavioral and psychological symptoms of dementia: an-open label, add-on, pilot study. J Alzheimers Dis. (2016) 51:15–9. doi: 10.3233/JAD-150915, PMID: , PMID: 26757043

[21] Sawicki SM Hernandez C Laiteerapong N Zahradnik EK. The use of dispensary-obtained tetrahydrocannabinol as a treatment for neuropsychiatric symptoms of dementia. J Clin Psychiatry. (2023) 84:23m14791. doi: 10.4088/JCP.23m14791, PMID: , PMID: 37728481

[22] Lubman DI Cheetham A Yucel M. Cannabis and adolescent brain development. Pharmacol Ther. (2015) 148:1–16. doi: 10.1016/j.pharmthera.2014.11.009, PMID: , PMID: 25460036

[23] Manuel I Gonzalez de San Roman E Giralt MT Ferrer I Rodriguez-Puertas R. Type-1 cannabinoid receptor activity during Alzheimer's disease progression. J Alzheimers Dis. (2014) 42:761–6. doi: 10.3233/JAD-140492, PMID: , PMID: 24946872

[24] Scott JC. Impact of adolescent cannabis use on neurocognitive and brain development. Child Adolesc Psychiatr Clin N Am. (2023) 32:21–42. doi: 10.1016/j.chc.2022.06.002, PMID: , PMID: 36410904

